# Differences in Challenges to Using Telehealth Among Older Adult Video and Telephone Users With Frailty: Retrospective Observational Study

**DOI:** 10.2196/69437

**Published:** 2025-08-29

**Authors:** Saanvi Lamba, Shirley Li, Avi Lamba, Fei Tang, Willy Marcos Valencia, Stuti Dang, Elizabeth Dole Center of Excellence for Veteran and Caregiver Research

**Affiliations:** 1 Flint Hill School, Oakton, VA, United States; 2 Keck School of Medicine, University of Southern California, Los Angeles, CA, United States; 3 University of Rochester, Rochester, NY, United States; 4 Geriatric Research Education and Clinical Center, Miami Veterans Affairs Healthcare System, 1201 NW 16th Street, Miami, FL, 33125, United States, 1 3055753388; 5 Endocrinology & Metabolism Institute, Cleveland Clinic Foundation, Center for Geriatric Medicine, Cleveland, OH, United States; 6 The Elizabeth Dole Center of Excellence for Veteran and Caregiver Research, San Antonio, TX, United States; 7 Miller School of Medicine, University of Miami, Miami, FL, United States

**Keywords:** telehealth, video visits, telephone visits, older adults, frailty, technology

## Abstract

**Background:**

The shift to video care during the COVID-19 pandemic exacerbated disparities in health care access, especially among high-need, high-risk older adults with frailty.

**Objective:**

The objective of this study was to quantify the ability of high-need, high-risk older veterans to use video visits for health care and identify factors associated with successful video visit completion.

**Methods:**

Veterans in a Veterans Affairs Frailty Intervention and Treatment (FIT) clinic underwent a physical, functional, psychological, social, and technology assessment at baseline. During the pandemic, the FIT clinic switched to televisits. We sorted patients into 4 groups: video visit, telephone visit, reached but no visit, and unable to contact. We performed a *t* test to compare normal variables and a chi-square test to compare categorical variables to identify factors associated with completing a video visit versus a telephone visit.

**Results:**

We attempted to contact 110 patients from the FIT clinic. They were 73.5 (SD 5.6) years old on average. A total of 46 (41.8%) patients were White, 46 (41.8%) patients were Black, and 17 (15.5%) patients were Hispanic. Sixty-seven (60.9%) patients had at least some college education, and 49 (44.5%) patients were very confident in filling out medical forms. Of the 110 patients, 72 patients were reached, and 65 patients agreed to a televisit. Of the 65 patients who agreed to a televisit, 19 (29.2%) patients completed a video visit, while 37 (57%) completed a telephone visit. A total of 19 patients out of 25 (76%) of those who scheduled a video visit completed it successfully. Compared with those who completed a telephone visit, veterans who completed a video visit were more likely to have access to a computer with a camera, microphone, and high-speed internet/data plan, as well as the ability to use email and be confident in internet use. They were more likely to have a higher health literacy score and be cognitively intact with a Montreal Cognitive Assessment score of ≥26, and were less likely to experience issues with walking, stepping, and balance.

**Conclusions:**

Our study found that completing a video visit requires access to technology, the ability to use it, and a willingness to do so. Among older veterans with frailty, only a quarter completed a video visit, and this group comprised patients who already had access to video-capable technology, used it, and were comfortable with it. They were also more often physically and cognitively intact compared with those who used telephone visits. Strategies to expand the use of video visits in the care of older adults include screening older adults to identify individuals most likely to succeed and increasing access to simple home telehealth technology.

## Introduction

Video-based telehealth emerged as a crucial care-delivery tool during the COVID-19 pandemic, offering potential benefits that include reducing not only infection risk, but also transportation needs and patient and caregiver time commitments, while maintaining continuity of care. The COVID-19 pandemic forced health care systems and patients who had not previously relied on telemedicine technology to use it to deliver care under “stay at home” orders.

The Department of Veterans Affairs Health Care System (VA) has consistently been a leader in expanding technology use in health care and continued to build upon its telemedicine expansion during the pandemic [1,2]. During this period, veterans were able to access their VA providers using the camera on their smartphone, computer, or tablet, along with an email address, using the VA Video Connect platform. The VA adjusted its Health Insurance Portability and Accountability Act (HIPAA) compliance requirements during the early days of the pandemic to facilitate connections with patients on non-public-facing technology when VA Video Connect was not accessible or at overcapacity. Apple FaceTime, Facebook Messenger video chat, Doximity, Google Hangouts video, and Skype were authorized for provider use [1,2].

Older adults with frailty stood to benefit from televisits, as they were at high risk for COVID-19 infection and poorer health outcomes [3]. However, several studies have shown that the uptake of televisits among older adults was rather heterogeneous [4-7]. To develop data-driven approaches for deploying telemedicine that improve access to care among complex older adults with frailty, it is essential to understand what proportion can successfully complete video visits and to identify the factors associated with their successful use.

The primary aim of this study was to evaluate the proportion of older adults with frailty who were able to complete a video visit for receiving health care. The secondary aim was to characterize the physical and psychosocial differences among older adults who were able to conduct video versus telephone visits for nonemergent reasons. This paper aims to contribute to the ongoing efforts to provide actionable data that can help leverage telemedicine as a means of increasing access to health care.

## Methods

### Ethical Considerations

This study was exempted by the Miami Veterans Affairs Institutional Review Board (Human Studies Subcommittee) and determined to be a quality improvement project (reference number 1360043) with minimal risk. Documentation of informed consent was not required. However, participation in the Frailty Intervention and Treatment (FIT) clinic was entirely voluntary, and participants could withdraw at any time. To ensure privacy and confidentiality, names, addresses, and Social Security numbers were deidentified, replaced with a unique code, and all study data was maintained behind a VA firewall. No compensation was provided.

### Geriatric Frailty Intervention and Treatment (FIT) Consultation Clinic

This retrospective observational study was part of a larger quality improvement study—the Geriatric FIT consultation clinic for older veterans with frailty in one urban VA medical center. Our Geriatric FIT clinic recruited veterans older than 65 years from a registry of high-need, high-risk (HNHR) veterans, who had been identified by the VA using population health tools and VA health care utilization data. HNHR veterans are medically complex, functionally impaired, and at the highest risk for adverse health outcomes, including hospitalization and long-term institutionalization. They also qualified for Medicare’s demonstration of Home-Based Primary Care (ie, Independence at Home) [8]. HNHR veterans are recommended for preemptive targeting to receive enhanced services and support, thereby preventing poor outcomes such as long-term institutional care and hospitalization. In this clinic, we conducted comprehensive geriatric and frailty assessments for older veterans with frailty, including a comprehensive baseline questionnaire to link them to needed services and support. We also offered recommendations to help mitigate frailty [9]. We followed patients for approximately 12 months and saw them approximately every 6 months in our consultative clinic, with the goal of completing 3 total visits per patient [8,9].

When society rapidly adopted social distancing at the beginning of the pandemic, the VA transitioned to televisits. Here, we analyze the completion of video versus telephone visits for the 6-month follow-up visits in the nonemergent FIT consultation clinic.

### Scheduling Patients for Home Telehealth Visits

Between March 30 and October 19, 2020, when care transitioned to telemedicine during the pandemic, we attempted to contact all 110 FIT clinic patients by telephone. We asked patients we reached if they would be interested in scheduling a follow-up televisit FIT clinic appointment. If a patient agreed to a televisit, we asked them about their access to technology, specifically, a device with a camera or microphone and internet at home. We had collected technology data during the baseline visit before the pandemic but gathered this information again since a change was possible in the interim. If they had the requisite technology, we asked about their willingness to do a video visit. Based on their preference, they were scheduled for a televisit via video or phone, and we documented the successful completion of the visit and the modality used. Successful completion meant finishing the entire consultation by video. If a video visit was initiated but had to be transitioned to telephone for any reason, including technical issues, it was categorized as a telephone visit.

We made 3 attempts to contact all 110 FIT clinic patients. Patients who were reached were informed that attendance was optional, as the FIT clinic served as a frailty consultation clinic rather than a primary care or problem-focused specialty clinic (eg, cardiology) requiring immediate attention. Ensuring needed follow-up and safety during the pandemic was conducted by patients’ primary care clinics through the VA-established pandemic protocol. It was not the role of the FIT consultation clinic. No new FIT clinic patients were recruited during this time.

Additionally, the coordinator called each scheduled patient the day before the televisit to remind them of the visit. If it was a video visit, the coordinator helped the patient set up, assisting them with installing and navigating the app if needed, and conducting a mock visit. During the mock visit, the coordinator completed a practice video visit, helping them program the microphone and camera, troubleshooting any issues that arose, and ensuring the patient could see and hear via the video technology. This assistance was also present during the visit.

### Data Collection During In-Person FIT Clinic Visit Before COVID-19

During their in-person FIT clinic visit before the pandemic, all patients had undergone a comprehensive geriatric assessment, including screening for cognitive impairment using a Montreal Cognitive Assessment (MoCA) [10], a frailty assessment including Short Physical Performance Battery [11], and grip strength, which were all repeated during their follow-up visits every 6 months. They also completed a baseline questionnaire on demographics and physical, functional, psychological, social, and technology characteristics (detailed in Table 1). The questionnaire has been elaborated upon in prior publications [7,12]. We calculated a modified Rockwood Frailty Index for all patients, using the deficit accumulation model of frailty with a count of 40 deficits in the denominator. The scoring for the deficits considered the severity of the current disease and the ability to perform daily activities [13].

**Table 1.** Baseline frailty clinic questionnaire components collected before COVID-19 [7].

Indicator	Source	Details
Demographics		
Education	Study-specific	Highest level of education completed.
Health literacy	Single question to identify patients with inadequate health literacy [14]	Confidence filling medical forms. Scores ranged from 1 to 5, with a higher score indicating more confidence (a score of 5 was considered health literate).
Physical domain		
Frailty	5-Item FRAIL Scale [15]	The 5-item FRAIL scale includes fatigue, resistance, ambulation, illnesses, and weight loss. The final score ranges from 0 to 5 and represents frail (score 3‐5), prefrail (score 1‐2), and robust (score 0) health status.
Self-rated physical status	Self-rated physical status	Scores for self-rated physical status ranged from 1 to 10, with a higher score indicating better physical status.
General health	Modified from the Stanford Chronic Disease Self-Management Program Questionnaire	Self-rated general health: scores ranged from 1 to 5, with a higher score indicating better self-rated general health.
Functional domain		
Activities of daily living (ADL)	Barthel Index for ADLs [16]	Number of ADLs they have problems with, Barthel’s ADL score (range 0‐100) with a higher score indicating greater independence.
Instrumental activities of daily living (IADL)	Lawton Score for IADLs [17]	Number of IADLs they have problems with, Lawton’s IADL score (range 0‐8) with a higher score indicating greater independence.
Walking and falls	Study-specific	Issues with walking, stepping, and balance; assistive devices used; the number of falls in the past year
Homebound status	Determining homebound status using validated questions from the National Health and Aging Trends Study [18]	Individuals were categorized as homebound, semihomebound, and not homebound based on their responses to how often they left their home, how much help they had leaving their home, and how much difficulty they had leaving their home in the previous month, like the reference study.
Psychological domain		
Depression screen	Patient Health Questionnaire-2 (PHQ-2) [19]	PHQ-2 scores ranged from 0 to 6 (a score ≥3 is considered positive for the likelihood of depression).
Self-perception of aging	Attitude toward own aging - subscale of the Philadelphia Geriatric Center Morale Scale [20]	The 5-question scale (range 0‐5) was treated as a binary variable. For the first (“feeling worse as I get older”) and third (“Feeling useless as I get older”) questions on the scale, the responses “strongly disagree, disagree, somewhat disagree” were scored as 0, whereas the responses “somewhat agree, agree, strongly agree” were scored as 1. The responses to the second (“as much pep as last year”), fourth (“as happy as when I was younger”), and fifth (“things are better than I thought it would be”) questions were scored in a reverse manner. A higher score indicated a negative self-perception of aging.
Social domain		
Social support	Study-specific	Having a formal or informal caregiver.
Social isolation	Berkman-Syme Social Network Index [21]	Scoring was performed as the following: Married (no =0; yes =1); meeting and talking to close friends and relatives (less than 3 times a week =0; 3 or more times a week =1); participation in religious meetings or services (less than 4 times a year =0; 4 or more times a year =1); attend meetings of the clubs or organizations (never/does not belong =0, all the responses =1). Scores were summed: 0 or 1 being the most isolated category; and 2, 3, or 4 formed the other 3 categories of increasing social integration.
Technology domain		
Technology	Study-specific	Access to video-capable technology, internet, and email; My HealtheVet enrollment and use; willingness to video visits; friend or family member to assist with technology. Confidence using technology (computer, internet, and email): The Likert-type options for this ranged from 0 to 4, the responses were treated as a binary variable, and responses of either 3 (quite a bit) or 4 (extremely) were considered as being confident. Preferred mode of contact.

During COVID-19, when visits were telemedicine, patients underwent a blind MOCA [22,23] instead of a regular MOCA, and physical assessments for frailty, including the Short Physical Performance Battery [11], peak flow, and grip strength, were not performed. However, since we had comprehensive baseline data on all FIT clinic patients, we opted to use those baseline data for the data analyses, rather than using a blind MOCA and missing values for physical assessment.

We obtained additional data from VA records at baseline, including Care Assessment Needs scores (a VA measure for hospitalization and mortality risk) [24], JEN Frailty Index (a measure of frailty calculated using geriatric syndromes, functional deficits, and multimorbidity clusters, and used to predict nursing home admission) [25], Nosos score (a risk-adjusted score normalized to 1.0, meaning veterans would be considered relatively healthy, and therefore less costly, with a risk score less than 1.0) [26], Hierarchical Condition Categories [27], and the Area Deprivation Index, an established measure of socioeconomic disadvantage at the census tract level, from the Neighborhood Atlas.

### Data Collection When Scheduling FIT Clinic Follow-Up Visits During COVID-19

Data were collected on patients who were called and reached by telephone if they were interested in scheduling a televisit follow-up FIT clinic appointment, technology access (device with a camera and microphone, and high-speed internet), willingness to do a video visit (preference), type of televisit scheduled (video or phone), and completion of the televisit via video or phone.

### Data Collection After Telemedicine FIT Clinic Visit Completed During COVID-19

After completing the televisit FIT clinic appointment, patients were called by telephone to complete a 4-item patient satisfaction questionnaire regarding their experience with the televisit, conducted via video or telephone. They were asked to rate the following study-specific questions on a 3-point Likert scale with the options of disagree, neutral, or agree. (1) Overall, it was easy to communicate with the provider through the televisit. (2) I would rather use the televisit than travel to Miami to see the specialist in person. (3) Overall, I am satisfied with this televisit session. (4) I would recommend a televisit to others.

### Statistical Analysis

Patients were sorted into four groups as follows: (1) completed video visit; (2) completed telephone visit; (3) reached but no visit (veterans that were reached but refused to schedule [n=7], plus the group that was scheduled but not seen because they no-showed on the last day [n=9]); and (4) unable to contact (n=38). We compared the characteristics of the 4 groups, and then of those that successfully completed a video versus a telephone visit.

Descriptive characteristics were presented as frequencies (percentages) for categorical variables and means (SDs) for continuous variables. The Kolmogorov-Smirnov test was used to assess the normality of the distribution of the continuous variables. The Kruskal-Wallis rank sum test was used for comparing nonnormal continuous variables, and the *t* test was used for comparing normal variables. The chi-square test was used for comparing categorical variables. Furthermore, we compared the satisfaction differences between veterans who completed a video versus telephone visit, using the chi-square test. The *P* values were considered significant when less than .05. All the statistical analyses were performed with R (version 4.0.5; the R project for statistical computing).

## Results

### FIT Clinic Participants

We attempted to contact all 110 FIT clinic veterans between April and October 2020 to schedule a follow-up visit. The average age of the 72 veterans was 73.5 (SD 5.6) years. Forty-six (41.8%) veterans were White, 46 (41.8%) veterans were Black or African American, and 17 (15.5%) veterans were Hispanic or Latino. Sixty-seven (60.9%) veterans had at least some college education, and 49 (44.5%) veterans were very confident in filling out medical forms. On average, everyone had 3.2 (SD 1.8) major medical diagnoses. No new veterans were enrolled during this time. A total of 72 veterans were reached, 7 veterans declined, and 65 veterans were scheduled for a televisit based on their access to technology and preference.

### Televisits Completed

Of the 65 scheduled patients, 36 (55.4%) owned the requisite technology for a video visit. Of these 36 patients, 25 (38.5%) were willing and scheduled for a video visit, while 11 patients preferred a telephone visit. Of the 25 patients scheduled for a video visit, 19 (76.0%) successfully completed it. However, these 19 (29.2%) patients represented less than a third of the 65 scheduled patients. Six of the 25 scheduled (24.0%) video visit patients were unable to complete the video visit due to issues with their internet connection, camera, or microphone, and one patient faced trouble installing the app, so they switched to a telephone visit.

**Figure 1.**
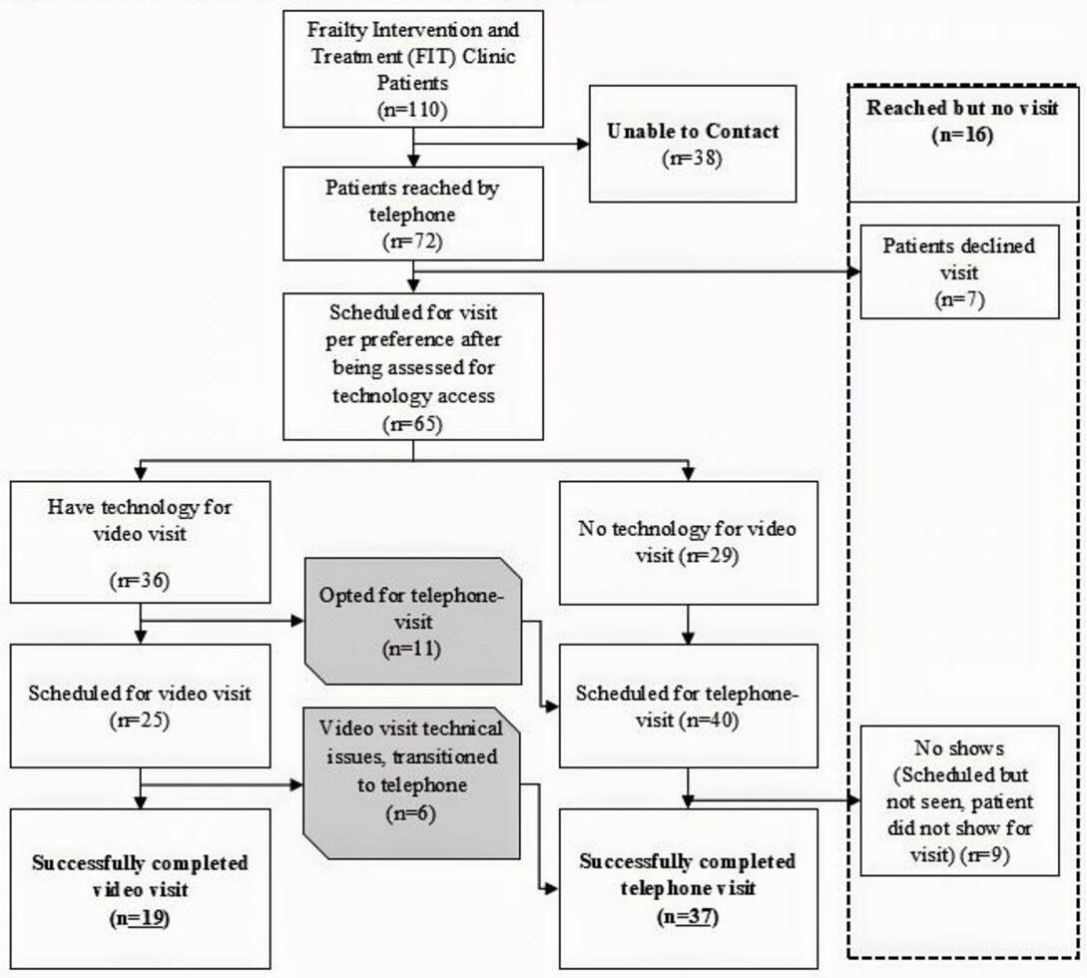
Flowchart showing FIT clinic patients who completed video and telephone visits. FIT: Frailty Intervention and Treatment.

Of the 65 patients scheduled, 40 (61.5%) were scheduled for a telephone visit, 29 (44.6%) did not have the requisite technology for a video visit, and 11 (16.9%) opted for a telephone visit despite having access to video-capable technology. However, 9 patients scheduled for a telephone visit were no-shows, and an additional 6 patients completed a telephone visit instead of a video visit due to technical challenges on the day of the visit. Therefore, 37 (56.9%) out of 65 patients ultimately completed a telephone visit successfully. Details are shown in Figure 1.

### Differences Between Subgroups

We compared the demographics, physical, functional, psychological, and technological characteristics of veterans across 4 groups: video visit completed (n=19), telephone visit completed (n=37), reached but no visit (n=16), and unable to contact (n=38). The results are shown in Tables2  3. There were a few significant differences between the 4 groups. It appears that veterans who completed a video visit were less frequently to have issues with walking, stepping, or balance (*P*=.03); more frequently to have caregivers (*P*=.02); score better on self-perception of aging (*P*=.04) (Table 2); and more frequently to have access to high-speed internet (*P*=.01), use email (*P*=.02), and be enrolled in My Health***e***Vet (*P*=.01) (Table 3). The “reached but no visit” group (n=17) was similar to the telephone-visit group in their access to high-speed internet and email, but somewhat less frequently enrolled in My HealtheVet, and none of them had reported having a caregiver (Tables2  3).

**Table 2.** Veterans’ sociodemographic characteristics by group (n=110).

	All patients (n=110)	Video visits completed (n=19)	Telephone visits (n=37)	Reached, no visit (n=16)	Unable to contact (n=38)	*P* values
Demographics						
Age (years), mean (SD)	73.4 (5.6)	73.3 (5.5)	73.3 (5.1)	74.5 (4.7)	73.0 (6.4)	.83
Age group (years), n (%)						.56
65‐69	26 (23.6)	4 (21.1)	10 (27.0)	2 (12.5)	10 (26.3)	
70‐74	45 (40.9)	9 (47.4)	15 (40.5)	5 (31.3)	16 (42.1)	
75‐79	22 (20.0)	3 (15.8)	7 (18.9)	7 (43.8)	5 (13.2)	
≥80	17 (15.5)	3 (15.8)	5 (13.5)	2 (12.5)	7 (18.4)	
Race or ethnicity, n (%)						.50
White, non-Hispanic	46 (41.8)	10 (52.6)	16 (43.2)	5 (31.3)	15 (39.5)	
Black, non-Hispanic	46 (41.8)	6 (31.6)	13 (35.1)	7 (43.8)	20 (52.6)	
Hispanic	17 (15.5)	3 (15.8)	8 (21.6)	3 (18.8)	3 (7.9)	
Other or unknown	1 (0.9)	0 (0.0)	0 (0.0)	1 (6.3)	0 (0.0)	
Health literacy, n (%)						
Very confident filling out medical forms (very =5)	49 (44.5)	13 (68.4)	14 (37.8)	5 (31.3)	17 (44.7)	.10
Average reported score (range 1‐5 ↑), mean (SD)	3.9 (1.3)	4.4 (1.1)	3.7 (1.3)	4.0 (1.1)	3.8 (1.3)	.17
Education, n (%)						.11
High school or less	40 (36.4)	2 (10.5)	14 (37.8)	5 (31.3)	19 (50.0)	
Some college to bachelor’s degree	54 (49.1)	14 (73.7)	18 (48.7)	9 (56.3)	13 (34.2)	
Master’s, doctoral, or professional degree	13 (11.8)	3 (15.8)	5 (13.5)	1 (6.3)	4 (10.5)	
Data from electronic health record, mean (SD)						
JFI^a^ ↓^b^	7.1 (1.4)	7.4 (1.4)	7.0 (1.3)	7.3 (1.5)	7.0 (1.3)	.73
HCC^c^ Conditions**↓**	5.3 (2.3)	5.2 (3.2)	5.1 (1.9)	5.6 (2.3)	5.5 (2.1)	.80
Care Assessment Needs (CAN) Score **↓**	92.8 (7.4)	90.4 (10.1)	94.1 (4.4)	95.2 (5.2)	91.6 (8.8)	.13
Nosos**↓**	2.7 (2.8)	3.1 (4.6)	2.5 (2.2)	2.6 (2.6)	2.8 (2.1)	.91
Physical domain						
Modified Rockwood Frailty Index, mean (SD) **↓**	0.3 (0.4)	0.3 (0.1)	0.3 (0.1)	0.3 (0.1)	0.4 (0.6)	.41
Frail scale, n (%)						.24
Frail	34 (30.9)	3 (15.8)	12 (32.4)	2 (12.5)	17 (44.7)	
Prefrail	44 (40.0)	7 (36.8)	14 (37.8)	9 (56.3)	14 (36.8)	
Robust	20 (18.2)	5 (26.3)	7 (18.9)	4 (25.0)	4 (10.5)	
Missing	12 (10.9)	4 (21.1)	4 (10.8)	1 (6.3)	3 (7.9)	
Self-rated physical status (range 1‐10), mean (SD)	5.9 (1.9)	6.4 (1.5)	5.8 (1.8)	6.3 (1.8)	5.4 (2.2)	.25
General health (range 1‐5), n (%)						.92
Very good or good	36 (32.7)	8 (42.1)	11 (29.7)	5 (31.3)	12 (31.6)	
Average	52 (47.3)	7 (36.8)	18 (48.7)	9 (56.3)	18 (47.4)	
Bad or very bad	21 (19.1)	3 (15.8)	8 (21.6)	2 (12.5)	8 (21.1)	
Functional domain, mean (SD)						
Activities of daily living (ADL) score (range 0‐100 ↑)	89.5 (14.6)	91.9 (14.5)	89.7 (13.0)	91.3 (11.7)	86.9 (17.4)	.62
Number of ADL Deficits	1.5 (2.0)	1.3 (2.2)	1.4 (1.5)	1.3 (1.7)	1.8 (2.3)	.68
Instrumental Activities of Daily Living (IADL) score (range 0‐8 ↑)	6.6 (1.5)	6.5 (1.7)	6.9 (1.5)	6.9 (1.0)	6.3 (1.6)	.38
IADL deficits, mean (SD)	1.4 (1.6)	1.5 (1.7)	1.1 (1.5)	1.0 (1.0)	1.7 (1.8)	.30
Issues with walking, stepping, or balance, n (%)	80 (72.7)	10 (52.6)	31 (83.8)	9 (56.3)	30 (79.0)	.03
Falls (1 or more), n (%)	65 (59.1)	9 (47.4)	23 (62.2)	9 (56.3)	24 (63.2)	.67
Assistive devices, n (%)						.92
None	47 (42.7)	10 (52.6)	16 (43.2)	7 (43.8)	14 (36.8)	
Cane	31 (28.2)	4 (21.1)	10 (27.0)	6 (37.5)	11 (29.0)	
Walker	27 (24.6)	4 (21.1)	10 (27.0)	3 (18.8)	10 (26.3)	
Wheelchair	4 (3.6)	0 (0.0)	1 (2.7)	0 (0.0)	3 (7.9)	
Homebound status, n (%)						.31
Completely homebound	7 (6.4)	1 (5.3)	1 (2.7)	2 (12.5)	3 (7.9)	
Semihomebound	21 (19.1)	1 (5.3)	8 (21.6)	2 (12.5)	10 (26.3)	
Not homebound	71 (64.6)	15 (79.0)	26 (70.3)	10 (62.5)	20 (52.6)	
Psychological domain						
Patient Health Questionnaire (PHQ-2) positive (score ≥3; range 0‐5), n (%)	22 (20.0)	4 (21.1)	5 (13.5)	1 (6.3)	12 (31.6)	.11
MoCA^d^ scores, mean (SD) ↑	21.9 (4.0)	23.3 (4.5)	21.8 (3.5)	21.8 (3.9)	21.3 (4.1)	.39
MoCA scores, n (%)						.11
≥26 (Normal cognitive health)	21 (19.1)	8 (42.1)	4 (10.8)	3 (18.8)	6 (15.8)	
18‐25 (Mild cognitive impairment)	70 (63.6)	8 (42.1)	28 (75.7)	10 (62.5)	24 (63.2)	
≤17 (Moderate to severe cognitive impairment)	17 (15.5)	3 (15.8)	4 (10.8)	2 (12.5)	8 (21.1)	
Missing	2 (1.8)	0 (0.0)	1 (5.3)	1 (6.3)	0 (0.0)	
Self-perception of aging scale (range 0‐5**↓**), mean (SD)	2.8 (1.5)	2.2 (1.5)	2.9 (1.6)	2.4 (1.3)	3.3 (1.4)	.04
Social domain						
Area Deprivation Index, n (%)						.64
1‐25	13 (11.8)	4 (21.1)	3 (8.1)	0 (0.0)	6 (15.8)	
26‐50	20 (18.2)	3 (15.8)	10 (27.0)	3 (18.8)	4 (10.5)	
51‐75	21 (19.1)	2 (10.5)	7 (18.9)	5 (31.3)	7 (18.4)	
76‐100	15 (13.6)	2 (10.5)	5 (13.5)	2 (12.5)	6 (15.8)	
NULL	41 (37.3)	8 (42.1)	12 (32.4)	6 (37.5)	15 (39.4)	
Caregiver status, n (%)						.002
No caregiver	72 (65.5)	9 (47.4)	26 (70.3)	16 (100.0)	21 (55.3)	
Caregiver	37 (33.6)	10 (52.6)	11 (29.7)	0 (0.0)	16 (42.1)	
Social Network Index (SNI) (range 0‐4 ↑), mean (SD)	1.9 (1.1)	2.1 (1.0)	2.0 (1.1)	2.0 (1.4)	1.6 (1.1)	.41
Transportation						
Commuting time (minutes), n (%)						.07
>120	3 (2.7)	0 (0.0)	3 (8.1)	0 (0.0)	0 (0.0)	
60‐120	34 (30.9)	5 (26.3)	9 (24.3)	7 (43.8)	13 (34.2)	
30‐59	45 (40.9)	7 (36.8)	19 (51.4)	2 (12.5)	17 (44.7)	
<30	27 (24.6)	6 (31.6)	6 (16.2)	7 (43.8)	8 (21.1)	

a JFI: JEN Frailty Index.

b The arrows depict the direction that indicates a better score for that assessment.

c HCC: Hierarchical Condition Categories.

d MoCA: Montreal Cognitive Assessment.

**Table 3.** Technology access, ability, and willingness from the video-visit checklist (n=110).

	All patients (n=110)	Video visits completed (n=19)	Telephone visits (n=37)	Reached, no visit (n=16)	Unable to contact (n=38)	*P* values
Technology access, n (%)						
Access to a device (computer, smartphone, or tablet)	50 (45.5)	13 (68.4)	13 (35.1)	6 (37.5)	18 (47.4)	.11
Access to high-speed internet or data plan	22 (20.0)	9 (47.4)	6 (16.2)	3 (18.8)	4 (10.5)	.01^a^
Technology ability, n (%)						
Confident in using computer	24 (21.8)	6 (31.6)	8 (21.6)	4 (25.0)	6 (15.8)	.58
Confident in using the internet	27 (24.6)	7 (36.8)	9 (24.3)	4 (25.0)	7 (18.4)	.51
Confident in using email	36 (32.7)	7 (36.8)	13 (35.1)	6 (37.5)	10 (26.3)	.77
Have access to email	56 (50.9)	15 (79.0)	20 (54.1)	7 (43.8)	14 (36.8)	.02^a^
Able to do internet search	61 (55.5)	14 (73.7)	20 (54.1)	8 (50.0)	19 (50.0)	.36
Enrolled in My HealtheVet	48 (43.6)	13 (68.4)	19 (51.4)	6 (37.5)	10 (26.3)	.01^a^
Technology willingness, n (%)						
Willing to use videoconference	60 (54.6)	13 (68.4)	18 (48.7)	8 (50.0)	21 (55.3)	.54
Interest in televisit versus in-person VA^b^ visit	56 (50.9)	14 (73.7)	19 (51.4)	8 (50.0)	15 (39.5)	.16
Caregiver support, n (%)						
Anyone to assist with video-visit	60 (61.2)	13 (68.4)	19 (51.4)	6 (40.0)	22 (81.5)	.30
The preferred way to be contacted by VA, n (%)						.39
Home phone	27 (24.6)	3 (15.8)	11 (29.7)	3 (18.8)	10 (26.3)	
Cellphone	63 (57.3)	9 (47.4)	20 (54.1)	9 (56.3)	25 (65.8)	
Internet	5 (4.4)	1 (5.3)	1 (2.7)	2 (12.5)	1 (2.6)	
Mail	14 (12.7)	5 (26.3)	5 (13.5)	2 (12.5)	2 (5.2)	

a The *P* values were considered significant when less than .05.

b VA: Veterans Affairs.

We characterized the subgroup that completed their visits via telephone despite having access to technology. This included the 11 video-capable patients who chose telephone and the 6 patients who had to switch to telephone visits on the last day (n=17). Additionally, we characterized the group that had a telephone preference or video problem (n=17). These included individuals who preferred telephone visits despite having access to technology (n=11) and those who experienced video technology/connectivity problems on the day of the visit and were subsequently converted to telephone visits (n=6). None of these subgroups differed significantly from the other FIT clinic participants. Their detailed characteristics are presented in Tables S1 and S2 in Multimedia Appendix 1, respectively.

We then compared the differences in characteristics between veterans who completed video visits versus those who completed telephone visits. Only significant differences are shown in Table 4. Veterans who completed a video visit were more likely to have access to a computer with a camera and microphone (*P*=.001) and high-speed internet/data plan (*P*=.03), use email (*P*=.02), and use (*P*=.05). They were more likely to have a higher health literacy score (*P*=.04), be cognitively intact with a MoCA score of ≥26 (*P*=.02), and be less likely to have issues with walking, stepping, and balance (*P*=.03).

**Table 4.** Differences among veterans who completed video versus telephone visits. Only statistically significant differences between the 2 groups are shown.

	Video visits completed (n=19)	Telephone visits (n=37)	*P* values
Technology access and ability, n (%)			
Access to a computer with camera and microphone	16 (89.5)	10 (27.0)	*<*.001
High-speed internet or data plan	9 (47.4)	6 (16.2)	.03
Have access to email	17 (89.5)	20 (54.1)	.02
Confidence in internet use, n (%)			.05
Extremely/Quite a bit	7 (36.8)	9 (24.3)	
Somewhat/little/not at all	5 (26.3)	22 (59.5)	
Did not answer	7 (36.8)	6 (16.2)	
Health literacy			
Average reported score, mean (SD) ↑	4.4 (1.1)	3.7 (1.3)	.04
Cognition			
MoCA^a^ scores, n (%)			.02
≥26	8 (42.1)	4 (10.8)	
25 or less	11(57.9)	32 (86.5)	
Functional domain (mobility)			
Issues with walking, stepping, or balance, n (%)	10 (52.6)	31 (83.8)	.03

a MoCA: Montreal Cognitive Assessment.

### Satisfaction of Veterans Who Completed Video- Versus Telephone-Visit

A patient satisfaction questionnaire regarding their televisit experience was completed by 17 (89.47%) patients who had a video visit and 36 (97.29%) patients who had a telephone visit. There was no statistically significant difference among the video versus the telephone visit group in those who agreed regarding ease of communication with the provider (64.7% vs 72.2%), preference for a televisit rather than traveling to see a specialist in person (41.2% vs 22.2%), and satisfaction with the televisit session (70.6% vs 63.9%). However, a significantly greater proportion of patients who completed a video visit (n=13, 76.5%) said they would recommend a televisit when compared with those who completed a telephone visit (n=18, 50.0%; *P*=.03; Table S3 in Multimedia Appendix 1).

## Discussion

### Principal Findings

Our objective was to analyze the ability of highly vulnerable, older veterans with frailty to use video visits for health care. Our study shows that only a quarter of older HNHR veterans had the technology access, willingness, and ability to complete a video visit successfully. Patients who completed a video visit were more likely to have access to technology, already use email, and be confident in their use of technology. They were more likely to be health literate and be more cognitively and functionally intact compared with those who completed a telephone visit. However, these data provide a new perspective by comparing the type of appointment a veteran opted for, either as a preference or what was possible for them, and by quantifying the ability of HNHR older veterans to complete video visits successfully. The majority (three-quarters) of the patients who scheduled a video visit were able to complete it. The data also offer insight into potential screening questions to identify older adults who frequently participate successfully in video visits, including inquiring about access to a video-capable device, internet connectivity, patient comfort with technology, preferences, and cognitive abilities.

Our study aligns with existing research and confirms that among older adults with frailty, completing a video visit requires access to technology, a willingness or preference to use it, and the ability to use it effectively. Additionally, we found that technology adoption is not uniform—a small proportion of patients who had access to technology for video visits still preferred those by telephone. Many older patients with the requisite technological devices enabled for televisits may have concerns about digital literacy [28] or might be unable or unwilling to install video-capable apps onto their smartphones or tablets due to inadequate technology or difficulty with technology [4]. They may also be reluctant to participate in video visits due to concerns about their inability to communicate effectively over video, for reasons including cognitive or sensory impairment [29] lack of comfort with technology, or personal preference for in-person visits [30] Personal preference for in-person visits is seen in the satisfaction data from our project as well, wherein even though participants were largely pleased with the ease and quality of the video and telephone visits, they still had an overwhelming preference for in-person visits (almost 60% among our video visit and 80% among telephone visit participants), corroborating prior studies that have shown that older adults have a strong preference for in-person visits, which is cited among the most significant barriers to adoption of e-health services [30,31].

Despite some drawbacks, telephone visits may be a more accessible and reasonable alternative to video visits for some patients, especially older adults who face barriers to video visits or prefer this method of communication. Telephone visits, which offer patient privacy, are more feasible at a population level and can serve as a backup when video visits are unavailable or fail. Patient outcomes, including patient satisfaction, are generally comparable between video and telephone visits, as observed in our study as well [32]. Thus, telephone visits may be a critical way to engage older adults, especially when video visits are not an option. A video visit is not appropriate for every clinical need, and neither is a telephone visit. It is also important to consider telephone visits as a strong alternative to video visits, especially when video visits are not feasible, and to use caregivers for video visits when possible. Future research should establish a process to identify clinical scenarios in which the telephone or video may offer greater utility and develop hybrid models that combine in-person, video, and telephone visits, best suited to specific scenarios and patient types. Making telephone visits available as a modality of telemedicine should be an option while acknowledging that video visits may offer higher-quality care in certain situations.

Prior literature has recognized that older adults are more likely to face digital barriers, so they are less likely to engage in video visits [33-35] and risk being left behind during the rapid transition to telemedicine [7,36]. This is exaggerated among older adults from a lower socioeconomic status [37], those who are unhoused [37], non-English speaking [38], and racial/ethnic minorities [39]. Therefore, steps have to be taken to make video visits more accessible and feasible to use [40]. Some of these challenges, such as access to devices and high-speed internet, may be addressed by programs such as the “Digital Divide consult” in the VA, which provides data-enabled tablets to veterans who lack the requisite video-capable technology. Initiatives that enable trained staff, volunteers, students, peers, or community health workers to provide in-home training or serve as tele-presenters for in-home video visits [41], along with the strategic involvement of family caregivers and friends when available [29,42], can help high-risk older adults with frailty navigate the complexities of devices and apps. These efforts may improve digital literacy and even willingness to participate in telemedicine. However, some challenges may not be able to be addressed [43], and programs such as home-based primary care and in-person visits may be necessary to address these issues.

Previous data support that provider outcomes, including accuracy of initial diagnosis, physician-related medical errors, and self-reported abilities to address patients’ concerns, are improved with video visits when compared with telephone [32]. These benefits may reflect the visual assessment capabilities of video visits [44]. Other advantages to video visits for providers include a better ability to engage patients and caregivers, as well as potentially gain insight into the patient’s living situation, safety, social support, and medication adherence. Disadvantages include, as in our case, a limited ability to perform a complete cognitive or physical assessment [5]. The preference for video visits among some physicians raises concerns about striking a balance between access and the quality of care.

Our study has several limitations. The study population consisted of US veterans, who are mostly male and receive care through one large urban medical VA center; for these reasons, the results may not be generalizable to other population groups. Moreover, the findings may not represent the digital disparities that rural veterans may experience, as they are traditional users of telemedicine at the VA due to distance and transportation issues [45]. We do not identify ways to address preidentified technology limitations concretely, nor do we discuss attempts to intervene in response to the screenings or setbacks during the visit, nor document caregiver assistance during video visits. Another limitation is the time lapse between the baseline and follow-up FIT clinic visit. All patients underwent a detailed baseline assessment during their first FIT clinic visit (as tabulated in Table 1), along with the simultaneous extraction of relevant indices from their electronic medical records. Follow-up FIT clinic visits were scheduled 6 and 12 months after the baseline visit, and some patients’ situations may have changed during this period. Since there was a time-lapse of 6 to 12 months between the in-person baseline and the televisit follow-up appointment, it is possible that some patients’ situations and frailty status may have changed during this period, which is not accounted for in these analyses. However, since we had comprehensive baseline data on all FIT clinic patients, including those we could not reach during the COVID-19 pandemic, we opted to use these baseline data for this analysis, to address the limitations posed by variables not collected during televisits (physical performance metrics like grip strength or Short Physical Performance Battery), and the variables where in-person assessments (regular MoCA) were substituted with a blind MoCA, and to assess for differences between patients that were reached versus not reached during COVID. Since access to technology was one of the main considerations for scheduling a televisit, we reassessed technology access and willingness when calling to schedule the follow-up visit during the COVID-19 pandemic. Among the main limitations was the small sample size, which left us underpowered to find differences between the video visit and telephone visit groups. This study documents the use of telehealth for nonemergent health concerns. The nature of the visit may also influence patient preferences for telehealth.

On the other hand, one of the main strengths of this study was patient-level data focusing on an older group of patients with frailty, who are among the most frequently at risk of being left behind in this shift to telemedicine, and the systematic collection of technology access and digital literacy data, including on the group that was not reached during COVID. Extensive literature is available on the digital divide during the pandemic, focusing on the gap between those who have access to digital technologies and those who do not, as well as the characterization of groups that risk being left behind in the transition to telehealth [28,46,47]. However, there is less literature on the actual successful completion of video visits in a high-risk older adult population with frailty.

This paper adds evidence to the ongoing actionable data that may help leverage telemedicine as a means of increasing access to health care, especially for older adults with frailty and with multimorbidity and high complexity, in whom access to telehealth interventions is not only limited by barriers directly related to the multiple conditions that make them high-risk. Health care systems must continue efforts to understand the factors contributing to digital disparities and identify targeted interventions to address the technology gaps that disproportionately affect older high-risk adults. High-complexity, older patients are most likely to require additional resources and support. We may address disparities in video visit use by properly screening patients, addressing technology access and ability, and leveraging family caregivers among older adults to minimize their risk of being left behind in an increasingly virtual society and health care environment.

### Clinical Implications

With the shift to telehealth delivery of health care services, there is a need to ensure equitable telehealth access and to address existing technology gaps that disproportionately affect older high-risk adults. Only a quarter of high-risk older adults with frailty are able to successfully complete a video visit. Among those who schedule a video visit, however, three-quarters are able to complete it successfully. This highlights the importance of effective screening and patient identification to improve efficiency. Older adults who have access to technology already use technology, and they are more physically and cognitively intact and are more likely to complete a video visit. Addressing technology access and ability among older adults can increase access. Patients are equally satisfied with both video and telephone visits. Telephone visits may be a reasonable alternative for older adults who encounter barriers with video visits.

## Supplementary material

10.2196/69437 Multimedia Appendix 1 Supplementary tables of (1) characteristics of veterans reached but not visited, (2) characteristics of veterans who preferred telephone or had video technology problems and (3) patient satisfaction with visits via video or telephone.

## Abbreviations

FIT: Frailty Intervention and Treatment
HIPAA: Health Insurance Portability and Accountability Act
HNHR: high-need, high-risk
MoCA: Montreal Cognitive Assessment
VA: Veterans Affairs
