# Supplementary material for: Differences in Challenges to Using Telehealth Among Older Adult Video and Telephone Users With Frailty: Retrospective Observational Study
Source: J Med Internet Res. 2025 Aug 29;27:e69437. doi: 10.2196/69437 (PMC12396796; doi:10.2196/69437)
Supplement: Multimedia Appendix 1 [file jmir-v27-e69437-s001.docx]

**Table S1.** Characteristics of Veterans Reached But No Visit

|  | | **Reached, No Visit**  **(n=16)** | **Declined Visit**  **(n=7)** | **Scheduled but not seen**  **(n=9)** |
| --- | --- | --- | --- | --- |
| DEMOGRAPHICS | | | | |
| Age [mean ± SD] | | 74.5 ± 4.7 | 75.4 ±3.6 | 73.8 ± 5.5 |
| Age Group [n (%)] | | | | |
|  | 65-69 | 2 (12.5) | 0 (0.0) | 2 (22.2) |
|  | 70-74 | 5 (31.3) | 2 (28.6) | 3 (33.3) |
|  | 75-79 | 7 (43.8) | 4 (57.1) | 3 (33.3) |
|  | ≥80 | 2 (12.5) | 1 (14.3) | 1 (11.1) |
| Race/Ethnicity [n (%)] | | | | |
|  | White, Non-Hispanic | 5 (31.3) | 3 (42.9) | 2 (22.2) |
|  | Black, Non-Hispanic | 7 (43.8) | 4 (57.1) | 3 (33.3) |
|  | Hispanic | 3 (18.8) | 0 (0.0) | 3 (33.3) |
|  | Other/Unknown | 1 (6.3) | 0 (0.0) | 1 (11.1) |
| Health Literacy | | | | |
|  | Very confident filling out medical forms (very = 5); [n (%)] | 5 (31.3) | 3 (42.9) | 2 (22.2) |
|  | Average Reported Score [mean ± SD] ­ | 4.0 ± 1.1 | 4.0 ± 1.4 | 4.0 ± 0.8 |
| Education [n (%)] | | | | |
|  | High School or less | 5 (31.3) | 4 (57.1) | 1 (11.1) |
|  | Some College to Bachelor’s degree | 9 (56.3) | 3 (42.9) | 6 (66.7) |
|  | Master’s, Doctoral or professional degree | 1 (6.3) | 0 (0.0) | 1 (11.1) |
| DATA FROM ELECTRONIC HEALTH RECORD [mean ± SD] | | | | |
|  | JFI **↓** | 7.3 ± 1.5 | 7.6 ± 1.6 | 7.1 ± 1.4 |
|  | HCC Conditions**↓** | 5.6 ± 2.3 | 5.1 ± 1.8 | 6.0 ± 2.5 |
|  | Care Assessment Needs (CAN) Score **↓** | 95.2 ± 5.2 | 96.7 ± 3.2 | 94.0 ± 6.3 |
|  | Nosos**↓** | 2.6 ± 2.7 | 2.3 ± 1.7 | 2.9 ± 3.5 |
| PHYSICAL DOMAIN | | | | |
| Modified Rockwood Frailty Index, [mean ± SD] **↓** | | 0.3 ± 0.1 | 0.3 ± 0.1 | 0.3 ± 0.1 |
| Frail Scale | | | | |
|  | Frail | 2 (12.5) | 0 (0.0) | 2 (22.2) |
|  | Pre-frail | 9 (56.3) | 4 (57.1) | 5 (55.6) |
|  | Robust | 4 (25.0) | 3 (42.9) | 1 (11.1) |
|  | Missing | 1 (6.3) | 0 (0.0) | 1 (11.1) |
| Self-rated Physical Status [mean ± SD] ­ | | 6.3 ± 1.8 | 7.4 ± 1.4 | 5.4 ± 1.6 |
| General Health [n (%)] | |  |  |  |
|  | Very good/Good | 5 (31.3) | 4 (57.1) | 1 (11.1) |
|  | Average | 9 (56.3) | 2 (28.6) | 7 (77.8) |
|  | Bad/Very Bad | 2 (12.5) | 1 (14.3) | 1 (11.1) |
| FUNCTIONAL DOMAIN [mean ± SD] | | | | |
|  | Activities of Daily Living (ADL) score ­ | 91.3 ± 11.7 | 97.1 ± 4.9 | 86.3 ± 13.8 |
|  | Number of ADL Deficits [mean ± SD] | 1.3 ± 1.7 | 0.4 ± 0.8 | 2.0 ± 1.9 |
|  | Instrumental Activities of Daily Living (IADL) Score­ | 6.9 ± 1.0 | 7.3 ± 1.1 | 6.6 ± 0.9 |
| IADL deficits | | 1.0 ± 1.0 | 0.7 ± 1.1 | 1.4 ± 0.9 |
|  | Issues with walking, stepping, or balance, [n (%)] | 9 (56.3) | 2 (28.6) | 7 (77.8) |
|  | Falls (1 or more), [n (%)] | 9 (56.3) | 3 (42.9) | 6 (66.7) |
| Assistive Devices [n (%)] | | | | |
|  | None | 7 (43.8) | 4 (57.1) | 3 (33.3) |
|  | Cane | 6 (37.5) | 1 (14.3) | 5 (55.6) |
|  | Walker | 3 (18.8) | 2 (28.6) | 1 (11.1) |
|  | Wheelchair | 0 (0.0) | 0 (0.0) | 0 (0.0) |
| Homebound Status [n (%)] | | | | |
|  | Completely homebound | 2 (12.5) | 0 (0.0) | 2 (22.2) |
|  | Semi-homebound | 2 (12.5) | 0 (0.0) | 2 (22.2) |
|  | Not homebound | 10 (62.5) | 6 (85.7) | 4 (44.4) |
| PSYCHOLOGICAL DOMAIN | | | | |
|  | Patient Health Questionnaire (PHQ-2) positive [n (%)] | 1 (6.3) | 0 (0.0) | 1 (11.1) |
|  | MoCA Scores [mean ± SD] ­ | 21.8 ± 3.9 | 19.7 ± 4.4 | 23.6 ± 2.5 |
| MoCA Scores, n (%) | |  |  |  |
|  | 26 and above | 3 (18.8) | 1 (14.3) | 2 (22.2) |
|  | 18-25 | 10 (62.5) | 4 (57.1) | 5 (55.6) |
|  | 17 and below | 2 (12.5) | 2 (28.6) | 0 (0.0) |
|  | Missing | 1 (6.3) | 0 (0.0) | 1 (11.1) |
| Self-perception of Aging Scale, [mean ± SD] **↓**; range 0-5**¯** | | 2.4 ± 1.3 | 1.8 ± 1.2 | 2.9 ± 1.2 |
| SOCIAL DOMAIN | | | | |
| Area Deprivation Index [n (%)] | | | | |
|  | 1-25 | 0 (0.0) | 0 (0.0) | 0 (0.0) |
|  | 26-50 | 3 (18.8) | 0 (0.0) | 3 (33.3) |
|  | 51-75 | 5 (31.3) | 3 (42.9) | 2 (22.2) |
|  | 76-100 | 2 (12.5) | 1 (14.3) | 1 (11.1) |
|  | NULL | 6 (37.5) | 3 (42.9) | 3 (33.3) |
| Caregiver Status [n (%)] | | | | |
|  | No Caregiver | 16 (100.0) | 7 (100.0) | 9 (100.0) |
|  | Caregiver | 0 (0.0) | 0 (0.0) | 0 (0.0) |
| Social Network Index (SNI,) [mean ± SD]; range 0-4­ | | 2.0 ± 1.4 | 1.8 ± 1.5 | 2.1 ± 1.3 |
| TRANSPORTATION | |  |  |  |
| Commuting time, [n (%)] | | | | |
|  | >120 minutes | 0 (0.0) | 0 (0.0) | 0 (0.0) |
|  | 60–120 minutes | 7 (43.8) | 3 (42.9) | 4 (44.4) |
|  | 30–59 minutes | 2 (12.5) | 1 (14.3) | 1 (11.1) |
|  | <30 minutes | 7 (43.8) | 3 (42.9) | 4 (44.4) |

**Table S2.** Characteristics of Veterans who preferred Telephone or had Video Technology Problems

|  | **Telephone Preference or VoD Tech Problem**  **(N=17)** | **Preferred Telephone**  **(N=11)** | **VoD Tech Problem**  **(N=6)** |
| --- | --- | --- | --- |
| DEMOGRAPHICS | | | |
| Age [mean ± SD] | 72.8 ± 3.6 | 72.4 ± 4.3 | 73.7 ± 1.6 |
| Age Group [n (%)] | | |  |
| 65-69 | 3 (17.7) | 3 (27.3) | 0 (0.0) |
| 70-74 | 10 (58.8) | 5 (45.5) | 5 (83.3) |
| 75-79 | 4 (23.5) | 3 (27.3) | 1 (16.7) |
| ≥80 | 0 (0.0) | 0 (0.0) | 0 (0.0) |
| Race/Ethnicity [n (%)] | | |  |
| White, Non-Hispanic | 6 (35.3) | 3 (27.3) | 3 (50.0) |
| Black, Non-Hispanic | 6 (35.3) | 5 (45.5) | 1 (16.7) |
| Hispanic | 5 (29.4) | 3 (27.3) | 2 (33.3) |
| Other/Unknown | 0 (0.0) | 0 (0.0) | 0 (0.0) |
| Health Literacy | | |  |
| Very confident filling out medical forms (very = 5)  [n (%)] | 8 (47.1) | 6 (54.6) | 2 (33.3) |
| Average Reported Score [mean ± SD] ­ | 4.0 ± 1.1 | 4.2 ± 1.1 | 3.7 ± 1.2 |
| Education [n (%)] | | |  |
| High School or less | 4 (23.5) | 3 (27.3) | 1 (16.7) |
| Some College to Bachelor’s degree | 9 (52.9) | 5 (45.5) | 4 (66.7) |
| Master’s, Doctoral or professional degree | 4 (23.5) | 3 (27.3) | 1 (16.7) |
| PARAMETERS FROM ELECTRONIC HEALTH RECORD [mean ± SD] | | | |
| JFI **¯** | 6.9 ± 1.3 | 7.1 ± 1.6 | 6.7 ± 0.8 |
| HCC Conditions**¯** | 4.7 ± 2.2 | 5.1 ± 2.6 | 4.0 ± 0.6 |
| Care Assessment Needs (CAN) Score **¯** | 93.4 ± 5.1 | 94.1 ± 5.5 | 92.0 ± 4.4 |
| Nosos **¯** | 1.8 ± 1.4 | 2.0 ± 1.3 | 1.5 ± 1.5 |
| PHYSICAL DOMAIN | | | |
| Modified Rockwood Frailty Index [mean ± SD] ¯ | 0.3 ± 0.1 | 0.3 ± 0.1 | 0.3 ± 0.1 |
| Frail Scale | | |  |
| Frail | 5 (29.4) | 2 (18.2) | 3 (50.0) |
| Pre-frail | 6 (35.3) | 5 (45.5) | 1 (16.7) |
| Robust | 4 (23.5) | 3 (27.3) | 1 (16.7) |
| Missing | 1 (5.9) | 1 (9.1) | 1 (16.7) |
| Self-rated Physical Status [mean ± SD] ­ | 5.6 ± 2.2 | 6.2 ± 2.3 | 4.5 ± 1.4 |
| General Health [n (%)] | | |  |
| Very good/Good | 6 (35.3) | 6 (54.6) | 0 (0.0) |
| Average | 5 (29.4) | 3 (27.3) | 2 (33.3) |
| Bad/Very Bad | 6 (35.3) | 2 (18.2) | 4 (66.7) |
| FUNCTIONAL DOMAIN [mean ± SD] | | | |
| Activities of Daily Living (ADL) score ­ | 87.8 ± 12.2 | 88.0 ± 11.6 | 87.5 ± 14.4 |
| Number of ADL Deficits [mean ± SD] | 1.8 ± 1.5 | 1.6 ± 1.4 | 2.0 ± 1.8 |
| Instrumental Activities of Daily Living (IADL) Score ­ | 6.9 ± 1.3 | 6.8 ± 1.5 | 7.0 ± 0.9 |
| IADL deficits | 1.1 ± 1.3 | 1.2 ± 1.5 | 1.0 ± 0.9 |
| Issues with walking, stepping, or balance.  [n (%)] | 13 (76.5) | 8 (72.7) | 5 (83.3) |
| Falls (1 or more)  [n (%)] | 11 (64.7) | 6 (54.6) | 5 (83.3) |
| Assistive Devices [n (%)] | | |  |
| None | 5 (29.4) | 2 (18.2) | 3 (50.0) |
| Cane | 7 (41.2) | 6 (54.6) | 1 (16.7) |
| Walker | 5 (29.4) | 3 (27.3) | 2 (33.3) |
| Wheelchair | 0 (0.0) | 0 (0.0) | 0 (0.0) |
| Homebound Status [n (%)] | | |  |
| Completely homebound | 0 (0.0) | 0 (0.0) | 0 (0.0) |
| Semi-homebound | 4 (23.5) | 2 (18.2) | 2 (33.3) |
| Not homebound | 12 (70.6) | 8 (72.7) | 4 (66.7) |
| PSYCHOLOGICAL DOMAIN | | | |
| Patient Health Questionnaire (PHQ-2) positive [n (%)] | 1 (5.9) | 1 (9.1) | 0 (0.0) |
| MoCA Scores  [mean ± SD] ­ | 22.7 ± 2.9 | 22.6 ± 2.8 | 22.7 ± 3.3 |
| MoCA Scores n (%) | | |  |
| 26 and above | 2 (11.8) | 1 (9.1) | 1 (16.7) |
| 18-25 | 15 (88.2) | 10 (90.9) | 5 (83.3) |
| 17 and below | 0 (0.0) | 0 (0.0) | 0 (0.0) |
| Missing | 0 (0.0) | 0 (0.0) | 0 (0.0) |
| Self-perception of Aging Scale  [mean ± SD] **¯** | 2.6 ± 1.9 | 2.0 ± 1.9 | 3.7 ± 1.5 |
| SOCIAL DOMAIN | | | |
| Area Deprivation Index [n (%)] | | |  |
| 1-25 | 2 (11.8) | 0 (0.0) | 2 (33.3) |
| 26-50 | 3 (17.7) | 2 (18.2) | 1 (16.7) |
| 51-75 | 2 (11.8) | 2 (18.2) | 0 (0.0) |
| 76-100 | 3 (17.7) | 3 (27.3) | 0 (0.0) |
| NULL | 7 (41.2) | 4 (36.4) | 3 (50.0) |
| Caregiver Status [n (%)] | | |  |
| No Caregiver | 11 (64.7) | 6 (54.6) | 5 (83.3) |
| Caregiver | 6 (35.3) | 5 (45.5) | 1 (16.7) |
| Social Network Index (SNI)  [mean ± SD] ­ | 2.4 ± 1.2 | 2.4 ± 1.3 | 2.5 ± 0.9 |
| TRANSPORTATION | | | |
| Commuting time [n (%)] | | |  |
| >120 minutes | 2 (11.8) | 1 (9.1) | 1 (16.7) |
| 60–120 minutes | 5 (29.4) | 3 (27.3) | 2 (33.3) |
| 30–59 minutes | 8 (47.1) | 6 (54.6) | 2 (33.3) |
| <30 minutes | 2 (11.8) | 1 (9.1) | 1 (16.7) |

**Table S3**. Patient satisfaction with visits via video (N=17) or telephone (N=37).

|  | Overall, it was easy to communicate with the provider through the virtual visit | | I would rather use the virtual visit than travel to Miami to see the specialist in person. | | Overall, I am satisfied with this virtual visit session. | | I would recommend a virtual visit to others. | |
| --- | --- | --- | --- | --- | --- | --- | --- | --- |
|  | Video | Telephone | Video | Telephone | Video | Telephone | Video | Telephone |
| Disagree [n (%)] | 1 (5.9) | 1 (2.8) | 5 (29.4) | 11 (30.6) | 2 (11.8) | 1 (2.8) | 2 (11.8) | 1 (2.8) |
| Neutral [n (%)] | 5 (29.4) | 9 (25.0) | 5 (29.4) | 17 (47.2) | 3 (17.7) | 12 (33.3) | 2 (11.8) | 17 (47.2) |
| Agree [n (%)] | 11 (64.7) | 26 (72.2) | 7 (41.1) | 8 (22.2) | 12 (70.6) | 23 (63.9) | 13 (76.5) | 18 (50.0) |
| P-values | 0.79 | | 0.31 | | 0.26 | | 0.03 | |
